# Childbirth outcomes in rape-related pregnancies: a comparative study of women receiving care in a birthing room designed to promote person-centredness in eastern Democratic Republic of Congo

**DOI:** 10.1080/16549716.2025.2541535

**Published:** 2025-08-07

**Authors:** Urban Berg, Emile Mapatano, Maria Hogenäs, Marie Berg

**Affiliations:** a Institute of Clinical Sciences, Sahlgrenska Academy, University of Gothenburg, Gothenburg, Sweden; b Faculty of Medicine and Community Health, Evangelical University in Africa, Bukavu, Democratic Republic of the Congo; c Obstetrics and Gynecology, Panzi General Referral Hospital, Bukavu, Democratic Republic of the Congo; d Institute of Health and Care Sciences, Sahlgrenska Academy, University of Gothenburg, Gothenburg, Sweden

**Keywords:** Pregnancy after rape, obstetric outcomes, neonatal outcomes, childbirth experience, birth environment

## Abstract

**Background:**

There is limited knowledge regarding childbirth outcomes among women with pregnancies resulting from rape. At Panzi Hospital, a tertiary hospital in eastern Democratic Republic of Congo (DRC), a holistic care programme is provided for survivors of sexual violence.

**Objective:**

Explore childbirth outcomes among women classified as Robson Group 1 who received care in a birthing room designed to promote person-centredness, comparing those with rape-related pregnancies to other women.

**Methods:**

This study was conducted between March 2021 and July 2022, with consecutive collection of childbirth data from nulliparous women at term with a single foetus in cephalic presentation and spontaneous onset of labour, i.e. Robson Group 1. All participants received care in a new birthing room designed to promote person-centredness. Childbirth outcomes were compared between women with rape-related pregnancies (*n* = 159) and those without (*n* = 302).

**Results:**

No statistically significant differences in childbirth outcomes were observed. Among women with rape-related pregnancies, 81.8% had vaginal births, compared to 83.4% in the comparison group. Caesarean section rates were 18.2% and 16.6%, respectively. The childbirth experience was rated positively by 80.5% of women in the rape-related pregnancy group and 84.8% in the comparison group with scores of 8–10 on the modified Visual Analogue Scale for Overall Childbirth Experience (VAS-OCE, 0–10).

**Conclusions:**

Childbirth outcomes among women with rape-related pregnancies receiving care within a holistic programme were comparable to those of other pregnant women, when care was provided in a birthing room designed to promote person-centred care.

## Paper Context

- **Main findings:** Women with pregnancies resulting from rape, classified as Robson Group 1, receiving care in a birthing room adapted to promote person-centredness, had similar childbirth outcomes compared to other pregnant women in the same classification.
- **Added knowledge:** Women classified as Robson 1, whose pregnancies resulted from rape, may have good childbirth outcomes when being cared for in a more personally adapted birthing room and getting continuous perinatal preparation and support.
- **Global health impact for policy and action**: Special attention should be put on the care of women with pregnancy due to rape. Holistic care programmes and a person-centred approach may be used also in contexts with limited resources.

## Background

Sexual violence, a worldwide phenomenon threatening girls’ and women’s health, includes not only rape but any sexual act or attempt of a sexual act or any other unwanted act directed against a person’s sexuality conducted by any person regardless of their relationship to the victim [1,2]. About one-third of all women have experienced some kind of sexual violence, although the prevalence varies between contexts [3]. How often rape leads to pregnancy is not well known. A study from the United States found that pregnancy occurred in one in four women who had been raped [4], while a study from Ethiopia reported that 17% of female high school students who had been raped subsequently became pregnant [5].

Sexual violence is frequently used as a weapon in wars and conflicts, aimed at terrorising civilians and securing their access to valuable resources. This tactic partly explains the high prevalence in eastern Democratic Republic of Congo (DRC), where armed conflicts have been going on for decades [6]. According to a national household survey conducted in the DRC in 2007, 121 out of every 1,000 women of reproductive age reported having experienced rape, with 29 per 1,000 reporting such incidents within the preceding 12 months. However, a significantly higher number reported experiencing intimate partner sexual violence [7]. A cluster survey conducted in eastern DRC in 2010 found that 39.7% of women reported having experienced sexual violence at some point in their lives [8]. A recent study analysing data from four hospitals providing care to female survivors of sexual violence in Goma, the capital of North-Kivu, indicates a persistently high incidence. The majority of assaults were committed by strangers outside the victims’ homes and frequently involved threats with weapons [9].

Sexual violence has a negative impact on childbirth outcomes and reproductive health [10–17]. When experienced before and/or during pregnancy, it is associated with an increased risk of placental abruption, antepartum haemorrhage, stillbirth, preterm birth, and low birthweight. Other studies have found a higher risk for prolonged labour, planned or emergency CS as well as instrumental births, both in nulliparous and multiparous women with a history of sexual violence [12,14–16].

A systematic review of studies examining the consequences of war-related sexual violence identified post-traumatic stress disorder (PTSD) as the most frequently reported mental health outcome. The review also highlighted a high prevalence of stigmatisation by family and/or community members. The majority of the included studies were conducted in the DRC [18]. Another study from eastern DRC, based on interviews of women reporting rape-related pregnancies, found that social stigmatisation and rejection by the community significantly affected psychological well-being [19]. Women with a history of sexual violence have more fear of childbirth compared to those without such experiences [20,21] and demonstrate distinct needs and preferences regarding childbirth care [22,23]. Thus, the impact of sexual violence on pregnancy and childbirth is substantial.

The Panzi Hospital, a tertiary hospital located in Bukavu, in eastern DRC, serves a health zone with a population of half a million. It also functions as a specialised centre for victims of sexual violence, providing care through a holistic four-pillar model [24]. Over the past decades, more than 35,000 survivors of sexual violence have received care [25]. A recent observational study conducted over 2 years (2020–2022) at the hospital examined childbirth outcomes in 140 girls under the age of 18 with post-rape pregnancies. The study found a stillbirth rate of 2.9%, a preterm birth rate of 22.1%, and a Caesarean section (CS) rate of 30.7% [17].

Since birth is a sensitive neurohormonal, physiological, and psychological process, the surrounding environment also plays a role in influencing childbirth outcomes. A stressful and unfamiliar environment activates the sympathetic nervous system and inhibits the release of endogenous oxytocin, leading to weakened or ceased labour contractions, while a calm, private, and safe environment reduces stress and increases the release of endogenous oxytocin [26,27], which is favourable for a physiological vaginal birth.

As part of a research programme investigating the influence of the care environment, we conducted a quasi-experimental intervention study at the Panzi Hospital in 2021 to 2022 [28], where nulliparous women at term with one foetus in cephalic presentation and spontaneous onset of labour, i.e. Robson Group 1 [29], were included, except those with diagnosed intrauterine foetal death upon admission to the hospital. The study tested a new birthing room designed to enable calmness, safety, and person-centredness. Women were allocated to the new room when available and if occupied to the general birthing room. The study found that receiving care in the birthing room designed to promote person-centredness significantly promoted spontaneous vaginal birth without increasing complications. The rate of vaginal birth was 81.8% in the new room vs 70.3% in the group receiving care in the general room. The CS rate was 17.1%, compared to 28.4% (p-value 0.001) [28]. Over 25% of women in the study were pregnant due to rape. Most participants in this subgroup of post-rape pregnancies received care in the new birthing room (*n* = 159). This finding motivated us to conduct an analysis on available data and compare childbirth outcomes between women pregnant after rape with those of other women, who also received care in the new room.

Women pregnant after rape had followed a specific care programme based on holistic care principles to prepare them for childbirth and motherhood. The care programme included continuity of perinatal midwifery care, prenatal education, and empowerment, facilitating postnatal attachment between mother and child. The aim was to enhance competence and capacity to enable the pregnant women empowerment and control throughout pregnancy, birth, and the year after.

## Methods

### Objective

The objective was to explore childbirth outcomes among women classified as Robson Group 1, who received care in a birthing room designed to promote person-centeredness, comparing those whose pregnancies resulted from rape with other women.

### Setting, study design, and population

This is a secondary analysis of a subpart of data from the quasi-experimental intervention study including women classified as Robson Group 1 at the Panzi Hospital, in Bukavu in the eastern part of the DRC, between March 2021 and July 2022 [28]. This secondary analysis includes only women who were cared for in the new room (*n* = 461), comparing childbirth outcomes in the group of women with pregnancy after self-reported rape (*n* = 159, age 13–31 years) to women who were pregnant without a history of rape (*n* = 302, age 15–35 years). More details of patient characteristics are presented in Table 1.

**Table 1.** Maternal characteristics of women in Robson group 1: comparison between women pregnant after rape and other women.*

Variable		Women pregnant after rape *n* = 159	Other women *n* = 302
Maternal age, years	Mean Median	16.8 (2.5) 16 (13; 31)	22.5 (3,7) 22 (15; 35)
Height, cm	Mean Median	151.3 (4.3) 150 (130; 168)	154.9 (5.0) 155 (145; 172)
Cohabiting with partner		2 (1.3%)	292 (96.7%)
Educational level	Incomplete primary school (< 6 y) Primary school (6 y) Secondary school (12 y) University exam (≥3 y completed)	12 (7.5%) 130 (81.8%) 17 (10.7%) 0 (0.0%)	1 (0.3%) 70 (22.7%) 199 (65.9%) 32 (10.6%)
Prenatal visits ≥ 4		149 (93.7%)	285 (94.4%)

*For categorical variables n (%) is presented. For continuous variables Mean (SD)/Median (Min; Max)/n = is presented.

### Data and statistical analysis

Included variables are maternal characteristics, obstetric and neonatal outcomes, and the mother’s self-reported experiences of childbirth and fear 2 h after birth. These rating questions were available in Swahili and in French. The questions had undergone face validity test in the local context. All variables are listed in Tables 1 and 2.

**Table 2.** Obstetric and neonatal outcomes of women in Robson group 1: comparison between women pregnant after rape and other women.*

Variable	Women pregnant after rape *n* = 159	Other women *n* = 302	p-value
Accompanying person	15 (9.4%)	113 (37.4%)	0.0001
Augmentation with oxytocin	9 (5.7%)	30 (9.9%)	0.1581
Oxytocin at expulsion	31 (19.5%)	49 (16.2%)	0.4377
Episiotomy	106 (66.7%)	211 (69.9%)	0.5261
Spontaneous vaginal birth	128 (80.5%)	249 (82.4%)	0.6135
Instrumental vaginal birth	2 (1.3%)	3 (1.0%)	–
Caesarean section	29 (18.2%)	50 (16.6%)	0.6969
Manual placenta delivery	1 (0.6%)	1 (0.3%)	–
Bleeding > 500 ml	3 (1.9%)	4 (1.3%)	–
Birth weight, g	3055 (372)	3134 (397)	< 0.0001
Low birth weight (defined as < 2500 g)	10 (6.3%)	16 (5.3%)	0.6751
Apgar < 4 at 5 min	1 (0.6%)	1 (0.3%)	–
Apgar 4–6 at 5 min	5 (3.1%)	3 (1.0%)	0.1312
Apgar 7–10 at 5 min	153 (96.2%)	298 (98.7%)	0.1005
Skin to skin < 2 h	128 (80.5%)	258 (85.4%)	0.1856
Breastfeeding < 2 h	158 (99.4%)	301 (99.7%)	1.0
Fear of birth (defined as ≥ 6 on VAS 0–10)	97 (61.0%)	169 (56.0%)	0.3220
Overall childbirth experience Mean (SD) Overall childbirth experience Median (range)	9.08 (1.44) 10 (3–10)	9.18 (1.24) 10 (4–10)	0.4371
Mixed or Bad overall childbirth experience (1–7 on the VAS-OCE scale 1–10)	31 (19.5%)	46 (15.2%)	0.2932
Good overall childbirth experience (8-–0 on the VAS-OCE scale 1–10)	128 (80.5%)	256 (84.8%)	0.2932

*For categorical variables n (%) is presented. For comparison between groups Fisher’s Exact test 2-sided p-value was used for dichotomous variables. For continuous variables Mean value in grams and Standard deviation is presented.

For the variable fear and worry during childbirth, we used a modified Fear of Birth Scale (FOBS), which measures fear of birth by asking individuals during pregnancy questions regarding fear and worry about their approaching birth. The FOBS score ranges from 0 to 100, where a score ≥60 is defined as fear of childbirth [30]. The women were asked 2 h after birth to rate their fear during childbirth and their worry about giving birth again. In this study, we used a modified FOBS scale score ranged from 0 to 10. Thus, a rating of ≥6 corresponds to ≥60 on the original FOBS. According to a multicountry study, both original and modified FOBS score ranges had good to excellent reliability during pregnancy and postpartum [31].

The question assessing the overall childbirth experience was phrased as: ‘What was your overall experience of the childbirth?’ Women responded using a numeric rating scale from 1 (Very bad) to 10 (Very good), corresponding to the Visual Analogue Scale of Overall Childbirth Experience (VAS-OCE) [32]. Responses were analysed by calculating the mean, median, standard deviation, and range as measures of central tendency and dispersion. For dichotomous analysis, responses were grouped into two categories: VAS-OCE scores of 1–7 indicated a bad or mixed experience, while scores of 8–10 reflected a good overall experience [33].

Comparison between the two groups was performed using Fisher’s exact test for dichotomous variables and Fisher’s non-parametric permutation test for comparing independent means of continuous variables. The mean differences with 95% confidence intervals and p-values were the main results. All statistical tests were two-sided and conducted at the 5% significance level.

Study procedures were performed in accordance with the Declaration of Helsinki. Patient consent, confidentiality, and anonymity were respected. Ethical approval was given by the DRC’s National Ethical Committee of Public Health (CNES001/DPSK/192PP/2022).

## Results

Women whose pregnancies resulted from rape were younger (16.8 vs 22.5 years, *p* = 0.0001), shorter (151.3 vs 154.9 cm, *p* = 0.0001), had less education (see Table 1), and rarely lived with a partner (1.3% vs 96.7%). Nearly all in both groups attended more than four prenatal visits (93.7% vs 94.4%; Table 1).

The childbirth and neonatal outcomes are presented in Table 2. In women pregnant after rape, oxytocin was used for labour augmentation in 5.7% of cases compared to 9.9% in other women. Episiotomy was performed in a high proportion in both groups: 66.7% vs 69.9%. Vaginal birth was the most common mode of birth: 81.8% in women with pregnancies following rape and 83.4% in other women. Caesarean sections occurred in 18.2% and 16.6% of cases, respectively, while instrumental vaginal births were rare. Very few women had a postpartum haemorrhage defined as >500 ml, 1.9% in the post-rape group vs 1.3% in other women. Apgar score <7 after 5 min was 3.8% vs 1.3% in the groups with pregnancy after rape vs other women. Skin-to-skin contact between mother and newborn for at least 2 h was achieved in 80.5% in women with pregnancy after rape vs 85.4% in other women, and almost all newborns were breastfed within 2 h after birth in both groups. The fear of birth was 61% in women pregnant after rape and 56% in other women. The overall childbirth experience was good (8–10 on the VAS-OCE scale) in both groups (80.5% vs 84.8%), with no significant difference. Only one woman in each group rated the experience as bad (≤4). There were no statistically significant differences in any of these outcomes.

Two significant differences were found: only 9.4% of women pregnant after rape had an accompanying person versus 37.4% in other women (*p* = 0.0001), and their infants had lower birth weights (3055 g vs 3134 g, *p* < 0.0001).

## Discussion

This study compared childbirth outcomes among women with rape-related pregnancies to those of women without a history of rape. Women with rape-related pregnancies had undergone preparation through a holistic care programme during the antenatal period. Both groups received care in a new birthing room designed to promote person-centred care. The same healthcare staff provided care to both groups, following identical care principles.

There were significant differences in sociodemographic characteristics between the groups. Women who became pregnant following rape were younger, had lower levels of education, and were rarely cohabiting with a partner. These factors have previously been identified as risk factors for preterm birth [34]. In addition, women with pregnancies resulting from rape were shorter, which may increase the likelihood of caesarean section [35,36]. Data on body mass index (BMI) were not available, as weight was not routinely documented within the prenatal care programme. Nevertheless, obesity is relatively uncommon in this region of eastern DRC, with a prevalence of only 6%, according to a study examining the characteristics of women exposed to sexual violence.

The mode of birth was similar in both groups. Women pregnant after rape had spontaneous vaginal births in 80.5% vs 82.4% and a CS rate of 18.2% compared to 16.6% in other women. This is a highly encouraging finding, indicating – as also observed in the earlier quasi-experimental study [28] – that the intervention of a birthing room designed to promote person-centredness had a positive influence on the mode of birth. This effect was evident even among women pregnant as a result of rape, despite, or regardless of, their increased vulnerability.

A study from Colombia demonstrated that maternal age may influence the mode of birth, with a higher risk of caesarean section observed in very young mothers <15 years [36]. However, a large study from the United States found that adolescents aged 15–19 years had a lower risk of caesarean section compared to young adults [37]. As our data are limited, they do not allow us to statistically analyse the influence of age on mode of birth.

The rate of episiotomy was similarly high in both groups of women in our study, with approximately two-thirds undergoing the procedure. This likely reflects the standard hospital practice at the time of the study – namely, the liberal use of episiotomy in nulliparous women. A study from a comparable setting in the neighbouring country of Rwanda reported an episiotomy rate of 80.1% among nulliparous women, compared to 19.9% in multiparous women [38]. The routine use of episiotomy in nulliparas is not supported by current evidence and should only be performed based on clearly defined clinical criteria [39]. These findings highlight the need to review the indications for episiotomy at the hospital, especially in nulliparous women, and the study has already contributed to increased awareness about avoiding unnecessary use of the procedure.

A positive finding was that the practices of skin-to-skin contact between mother and child for up to 2 h and initiating breastfeeding within 2 h were implemented in the same high degree in both groups. This reflects that the previous quality improvement actions at the hospital, to promote ‘zero separation’ for at least up to 2 h after birth had a positive impact also in women pregnant after rape.

The neonatal data in our study were limited as it included observations only at the labour ward, not during the entire neonatal care period. The rate of severe neonatal asphyxia was very low, with just one newborn with Apgar <4 within 5 min in each group. A study from the same hospital focusing on adolescents with pregnancy after rape has reported 22.1% premature childbirths and 9.6% of neonatal asphyxia [6]. As neonatal outcomes are highly influenced by prematurity, and our study population included only pregnancies at term, this explains why neonatal asphyxia was rare. The birth weight was significantly lower in the group with pregnancy after rape (3055 g vs 3134 g), which may be due to the fact that low birth weight is more frequent in adolescent mothers [40]. A more relevant figure is the number of newborns with a birth weight of <2500 g, as it is defined as low birth weight (LBW) according to the World Health Organization and negatively associated with infantile survival [41]. In the post-rape pregnancy group, there were 10 (6.3%) newborns with LBW compared to 16 (5.3%) in the other group, a difference that was not statistically significant. According to the literature, low socioeconomic status and short body height of the mother are risk factors for low birth weight of the baby [42,43].

To measure women’s experiences of childbirth and of fear was not a routine at the hospital, and to our knowledge, not elsewhere in the DRC. The overall childbirth experience measured through the VAS-OCE scale was good (VAS 8–10) in both groups (80.5% and 84.8%). This result is slightly better compared to a study in Rwanda, including both primi- and multiparous women, where 77.5% rated their childbirth experience as good [33]. However, these results are not comparative, as, in the Rwandan study, the childbirth experience was scored 1 month after the childbirth, and in our study, 2 h after birth.

The fear of birth has been reported to be a main issue in women with a history of sexual violence [44]. In our study, using a modified version of the FOBS scale, fear of birth (≥6) was slightly higher in the group of women with pregnancy after rape, but not statistically significantly (61.0 vs 56.0%).

### Strengths and limitations

We acknowledge the methodological limitations of this study. It is a secondary analysis of data originally collected to investigate the impact of a newly designed birthing room on childbirth outcomes. Participants were classified into two groups based on their self-reported history of pregnancy resulting from rape. There is a potential risk of underreporting rape-related pregnancies among women who did not disclose such a history, particularly given that many were cohabiting and may have experienced concerns related to stigma or shame. Unfortunately, the influence of mental health on childbirth outcomes could not be evaluated in this study, as it was not assessed. In particular, routine screening for post-traumatic stress disorder (PTSD) was not undertaken.

A strength of this study lies in the comparison of outcomes among women within the same Robson classification, during the same period and in the same context and birthing environment, thereby reducing the influence of several potential confounding factors.

## Conclusion

The study found that women with pregnancies resulting from rape who received care in a birthing room designed to be more person-centred, had childbirth outcomes as favourable as those of other women Robson Group 1. A specialised perinatal care programme, incorporating continuity of midwifery care, prenatal education, and empowerment for women pregnant after rape, may have contributed to these positive outcomes.

The findings have clinical implications for the study hospital, which is currently constructing a new maternity unit where the labour ward will include birthing rooms designed to promote person-centred care. Similar birthing rooms could be implemented in other labour wards across the country, as well as in comparable settings elsewhere. The results may also influence the design of birthing environments and the organisation of maternity care, particularly with regard to the integration of person-centred approaches. Further research is needed on childbirth outcomes among women pregnant as a result of rape, particularly concerning how the birthing environment and model of care influence postnatal attachment and long-term experiences of motherhood.

## Supplementary Material

STROBE_checklist_255476645_R1.docx

## Data Availability

Data will be shared upon reasonable request.

## Preprint information

A preprint of a previous version of the manuscript submitted to BMC Pregnancy and Childbirth is accessible on Research square with the following link: https://doi.org/10.21203/rs.3.rs-5025425/v1.

The manuscript was withdrawn by the authors due to unacceptable delay in the review process.
